# Development of a high-throughput γ-H2AX assay based on imaging flow cytometry

**DOI:** 10.1186/s13014-019-1344-7

**Published:** 2019-08-22

**Authors:** Younghyun Lee, Qi Wang, Igor Shuryak, David J. Brenner, Helen C. Turner

**Affiliations:** 1 Center for Radiological Research, Columbia University Irving Medical Center, 630 West 168th St, New York, NY 10032 USA; 2 Present Address: Laboratory of Biological Dosimetry, National Radiation Emergency Medical Center, Korea Institute of Radiological and Medical Sciences, 75 Nowon-ro, Nowon-gu, Seoul, 01812 Republic of Korea

**Keywords:** Imaging flow cytometry, DNA repair kinetics, Human lymphocytes, High throughput, Radiation sensitivity, Ionizing radiation

## Abstract

**Background:**

Measurement of γ-H2AX foci levels in cells provides a sensitive and reliable method for quantitation of the radiation-induced DNA damage response. The objective of the present study was to develop a rapid, high-throughput γ-H2AX assay based on imaging flow cytometry (IFC) using the ImageStream®^X^ Mk II (ISX) platform to evaluate DNA double strand break (DSB) repair kinetics in human peripheral blood cells after exposure to ionizing irradiation.

**Methods:**

The γ-H2AX protocol was developed and optimized for small volumes (100 μL) of human blood in Matrix™ 96-tube format. Blood cell lymphocytes were identified and captured by ISX INSPIRE™ software and analyzed by Data Exploration and Analysis Software.

**Results:**

Dose- and time-dependent γ-H2AX levels corresponding to radiation exposure were measured at various time points over 24 h using the IFC system. γ-H2AX fluorescence intensity at 1 h after exposure, increased linearly with increasing radiation dose (*R*^2^ = 0.98) for the four human donors tested, whereas the dose response for the mean number of γ-H2AX foci/cell was not as robust (*R*^2^ = 0.81). Radiation-induced γ-H2AX levels rapidly increased within 30 min and reached a maximum by ~ 1 h, after which time there was fast decline by 6 h, followed by a much slower rate of disappearance up to 24 h. A mathematical approach for quantifying DNA repair kinetics using the rate of γ-H2AX decay (decay constant, K_dec_), and yield of residual unrepaired breaks (F_res_) demonstrated differences in individual repair capacity between the healthy donors.

**Conclusions:**

The results indicate that the IFC-based γ-H2AX protocol may provide a practical and high-throughput platform for measurements of individual global DNA DSB repair capacity which can facilitate precision medicine by predicting individual radiosensitivity and risk of developing adverse effects related to radiotherapy treatment.

**Electronic supplementary material:**

The online version of this article (10.1186/s13014-019-1344-7) contains supplementary material, which is available to authorized users.

## Background

Double Strand Breaks (DSBs) are one of the most important types of DNA damage. DSBs are more difficult to repair than many other lesions and their incorrect repair (e.g., misrejoining of broken DNA strands from different chromosomes) can result in cytotoxic or genomic alterations. Defects in the DNA repair machinery may increase cell vulnerability to DNA-damaging agents and accumulation of mutations in the genome, and could lead to the development of various disorders including cancers. Epidemiological evidence supports a strong association between global DSB repair capacity and cancer risk [1–3], radiation sensitivity [4, 5] and response to cancer therapy [6, 7]. The association between genetic defects in DNA repair and increased clinical radiosensitivity has been identified in many studies and used as a basis for the development of predictive assays for normal tissue toxicity [8].

Over the past decade, the γ-H2AX assay has been applied to a variety of cell types and tissues to correlate γ-H2AX levels with DNA damage and repair [9–13]. Following radiation exposure, histone H2AX is rapidly phosphorylated by ATM and/or DNA-PK kinases at or near the vicinity of DNA DSB sites to form γ-H2AX [14]. Immunolabeling of γ-H2AX provides a quantitative measurement and direct visualization of DSBs as fluorescent nuclear foci. At the cellular level, the kinetics of formation or loss of γ-H2AX foci may reflect the rate or efficiency of DSB repair [15]. The biphasic nature of DSB repair kinetics has been associated with different repair pathways that allow repair for a fast (initial few hours) and slow component (hours to days) of repair [16, 17]. Additionally, there is evidence that the DSBs assayed several hours after the initial radiation challenge that still remain unrepaired known as residual DNA damage, may be predictive of individual susceptibility to complex DNA lesions that can be lethal [18]. Current evidence suggests that there is a large inter-individual variation in DSB DNA repair capacity in lymphocytes from healthy individuals [19–21]. Further, clinical radiosensitivity is often linked to defects in DNA repair [5, 22, 23]. The capacity to repair DSB is therefore an important factor to consider in risk assessment, however studies to date are limited due to no large-scale prospective evidence or ability to conduct high-throughput phenotypic assays [24].

The objective of the present study was to develop a rapid, high-throughput γ-H2AX assay based on imaging flow cytometry (IFC) using the ImageStream®^X^ Mk II (ISX MKII) platform to evaluate DNA DSB repair kinetics in human peripheral blood cells after exposure to ionizing irradiation. Imaging flow cytometry is a relatively new technique which combines the speed of flow cytometry with the imaging capability of conventional microscopy [25–27]. It has been used to analyze cell death, apoptosis and immune response as an advanced method for fluorescence-based analysis of cellular morphology and heterogeneity [28–33]. Combining the strength of flow cytometry and conventional microscopy enables high-throughput characterization of cells on a microscopic scale [34]. This paper presents: 1) dose response curves based on γ-H2AX fluorescence intensity and foci number, 2) measurements of DNA repair kinetics up to 24 h after exposure to 4 Gy γ rays and, 3) a mathematical approach for modeling DSB rejoining kinetics using two key parameters a) rate of γ-H2AX decay, and b) yield of residual unrepaired breaks.

## Methods

### Blood collection and irradiation

Blood was collected by venipuncture in 5 mL lithium-heparinized Vacutainer® tubes (BD Vacutainer™, Franklin Lakes, NJ) from healthy adult donors (2 female, 2 male) with informed consent and approval by the Columbia University Medical Center Institutional Review Board (IRB protocol IRB-AAAE-2671). All donors were non-smokers in relatively good health at the time of donation with no obvious illnesses such as colds, flu, or infections and no known exposures to medical ionizing radiation within the last 12 months. Fresh blood aliquots (1 mL) were dispensed into 15 mL conical bottom tubes (Santa Cruz Biotechnology, Dallas, TX) and were irradiated with γ rays (0, 2 and 4 Gy) using a Gammacell® 40 ^137^Cesium irradiator (Atomic Energy of Canada, Ltd., Chalk River, ON). The blood sample tubes were placed on their side in the middle of the chamber and irradiated with a dose rate of 0.73 Gy/min [35]. The ^137^Cs irradiator was calibrated annually with TLDs and homogeneity of exposure across the sample volume was verified using EBT3 Gafchromic film with less than 2% variation within the sample (Ashland Advanced Materials, Gafchromic, Bridgewater, NJ).

### γ-H2AX assay immunolabeling protocol

Immediately after irradiation, 100 μl blood aliquots were transferred to 1.4 mL 2D Matrix™ microtubes (Thermo Scientific™, Waltham, MA) containing 900 μL RPMI 1640 culture medium (Gibco, Waltham, MA) supplemented with 15% FBS and 2% Penicillin and Streptomycin (all reagents from Invitrogen, Eugene, OR). The rack containing microtubes was placed into an incubator at 37 °C, 5% CO_2_ up to 24 h. At specific time points after irradiation (0.5, 1, 3, 6 and, 24 h), cultured blood samples were lysed and fixed with 1X Lyse/fix solution (BD Phosflow™^,^; BD Biosciences, San Jose, CA), washed with 1X phosphate buffered saline (PBS, Gibco, Gaithersburg, MD), suspended in 50% cold methanol, and stored at 4 °C for 24 h. Fixed cells were permeabilized with 0.1% Triton X-100 (Sigma-Aldrich, St. Louis, MO) at room temperature for 10 min and then incubated with Alexa Fluor® 488 Mouse anti-H2AX (pS139) antibody (clone N1–431, BD Pharmingen™, Franklin Lakes, NJ), diluted 1:1000 with 1% bovine serum albumin (BSA, Sigma-Aldrich, St. Louis, MO) at room temperature for 1 h, after which the samples were washed with 1X PBS and stained with 5 μM DRAQ5™ (Thermo Scientific™) at RT for a minimum of 5 min. All solution transferring or mixing in microtubes was performed using a 1.2-ml multichannel electronic pipet (Eppendorf Inc., Westbury, NY). All steps in the procedure were performed at room temperature (RT) and microtubes in racks were spun at 250×g for 3 min.

### Data acquisition and analysis on the ISX and IDEAS®

The 96-well plate of samples were transferred to the ImageStream®^X^ Mk II (ISX MKII) imaging flow cytometer (LUMINEX Corporation, Austin, Texas) for automated sample acquisition and captured using the ISX INSPIRE™ data acquisition software. Images of 5000–12,000 cells were acquired at 40x magnification using the 488 nm excitation laser at 200 mW: Bright field (BF) images were captured on channel 1, γ-H2AX immunostaining on channel 2, DRAQ5 images on channel 5 and side scatter on channel 6. Data was collected with only the Area feature applied in the BF channel, such that events with areas less than 60 pixels (15 μm^2^) were gated out in order to minimize the collection of small debris. For the compensation, irradiated blood cells were stained with γ-H2AX antibody or DRAQ5 only and captured using the 488 nm laser without brightfield illumination. The compensation coefficients were calculated automatically using the compensation wizard in the Image Data Exploration and Analysis Software (IDEAS) package (v6.2). To quantify the γ-H2AX expression levels, the viable lymphocytes population was gated for foci quantification and total γ-H2AX fluorescence intensity. Nuclear foci formation was identified using the spot counting wizard in IDEAS which automated identification and enumeration of foci. The geometric mean of the γ-H2AX fluorescence intensity of individual cells from each sample was analyzed. For the dose response curve, γ-H2AX foci and intensity levels were measured at 1 h post irradiation. All curves were generated using GraphPad Prism 7 (GraphPad software Inc., La Jolla, CA), and R^2^ value was calculated to assess goodness of fit of curves from linear regression analysis.

### Quantitative modeling of DNA repair kinetics

For the kinetic curves, γ-H2AX levels were measured at 0.5, 1, 3, 6 and, 24 h after 4 Gy irradiation. The data on γ-H2AX foci (F) at different time points (T) after irradiation were quantitatively modeled by the following equation, where F_bac_ is the background value prior to irradiation, F_res_ is the residual value remaining at long times (e.g. 24 h) after irradiation, K_prod_ is the constant for induction of foci by radiation, and K_dec_ is the constant for decay of foci after irradiation [20]:

1 $$ \mathrm{F}={\mathrm{F}}_{\mathrm{bac}}+{\mathrm{F}}_{\mathrm{res}}+{\mathrm{K}}_{\mathrm{prod}}\ T\ \mathit{\exp}\left(-{\mathrm{K}}_{\mathrm{dec}}\ T\ \right) $$

We used least squares fitting in Maple 2017 software (https://www.maplesoft.com/) as a practical approach for estimating K_dec_ and F_res_, involving curve fitting of each sample data set to Eq. (1). Thus, as we propose below we will use both the decay constant (K_dec_) and residual excess fluorescence intensity (F_res_) to describe each individual’s DNA DSB Repair Capacity.

## Results

### Development of IFC-based high throughput γ-H2AX assay

**Fig. 1:**
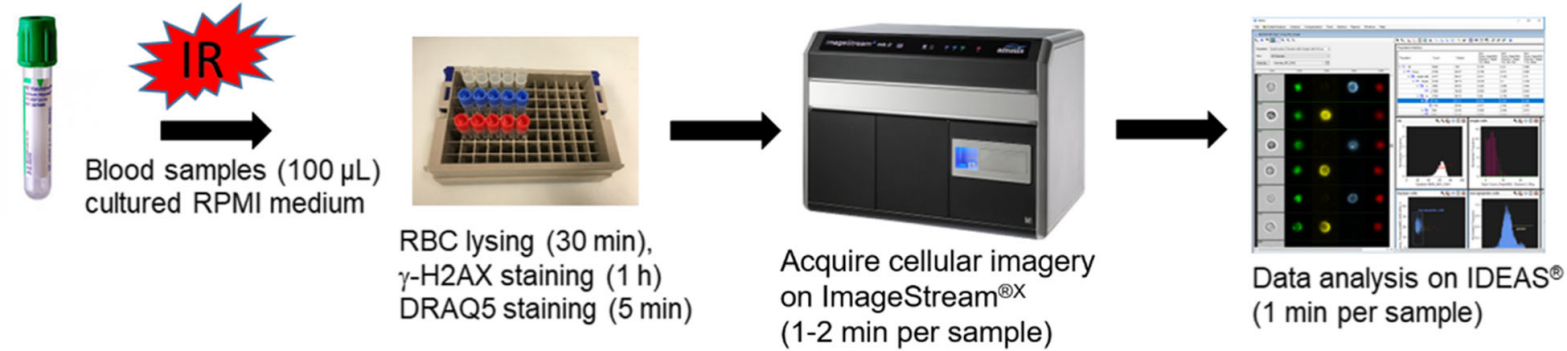
Development of a simple and fast γ-H2AX assay protocol. Fresh blood samples (100 μL) were prepared and cultured in RPMI medium following gamma irradiation. At specific time points up to 24 h after irradiation, whole blood samples were lysed, fixed and stained with γ-H2AX antibody and the nuclei were counter-stained with DRAQ5. Cellular imagery was automatically captured using the ISX INSPIRE™ software that controls the ImageStream®^X^ (ISX) Mark II imaging flow cytometer. All acquired imagery was analyzed by IDEAS® software

We have developed a simple and rapid IFC-based γ-H2AX protocol, comprised of the following four components: (1) Sample preparation of finger-stick sized blood samples (< 100 μL) in 96 well format, (2) Automated cellular image acquisition of immunofluorescent-labelled biomarkers using the ISX MKII system (3) Quantification of γ-H2AX biomarker levels using IDEAS and, (4) Quantitative modeling of DNA repair kinetics in peripheral blood lymphocytes. Figure 1 shows the schematic work flow for the IFC-based γ-H2AX protocol. In general, the immunolabeling protocol is less than 2 h while the acquisition and analysis of each sample (~ 3000 non-apoptotic human lymphocytes) can be finalized within 3 min.

### Quantification of γ-H2AX levels using IDEAS software

**Fig. 2:**
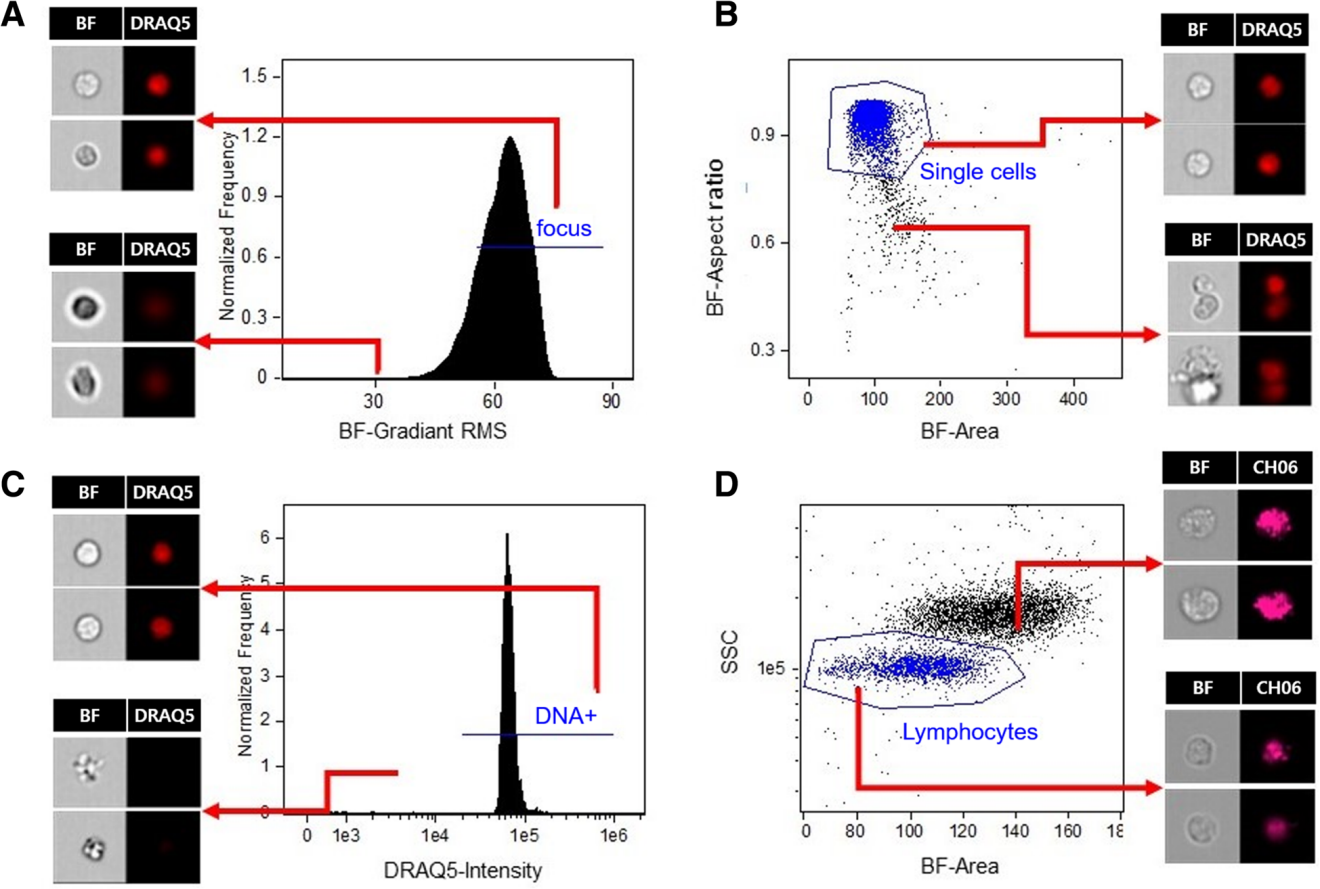
Gating strategy for assessing γ-H2AX levels in the IDEAS® software. **a** Using the Gradient RMS feature in the brightfield (BF) channel, which indicate sharpness of an image, cells with optimal focus were selected. **b** Using the area and aspect ratio features in the brightfield channel, single cells were selected and doublet events were removed. **c** DNA positive cells were selected based on DRAQ5 positivity and DNA negative cells were removed. **d** Lymphocytes were selected based on their size using the BF area and SSC intensity features

**Fig. 3:**
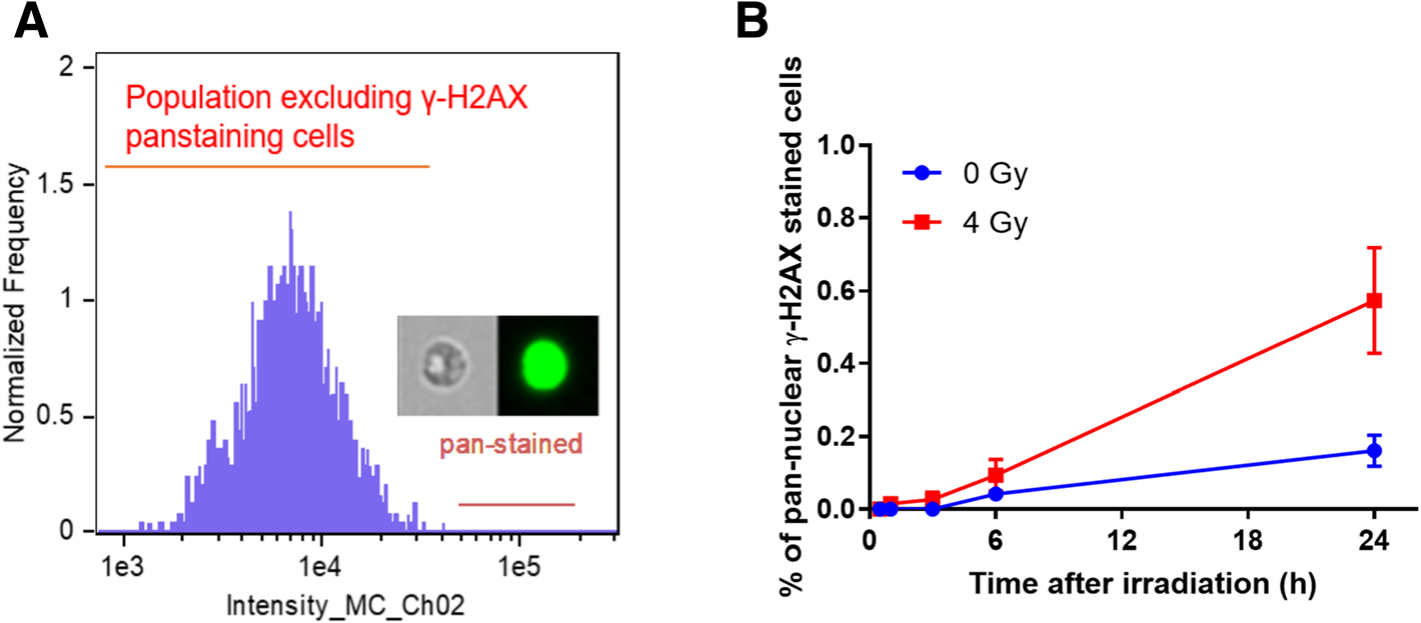
Percentages of pan-nuclear γ-H2AX stained cells increase with time in irradiated and non-irradiated cells. **a** Gating of pan-nuclear γ-H2AX stained cells. **b**. Percentages of pan-nuclear γ-H2AX stained cells as a function of increasing dose. The data is presented as mean ± SEM

Figure 2 shows the gating strategy to identify γ-H2AX levels in non-apoptotic human lymphocytes from the cell population. The focused cells were gated according to the gradient root mean squared (RMS) feature by visual inspection of cell images in the brightfield channel (Fig. 2a). Single cells were then selected from images according to their area and aspect ratio in the brightfield channel (Fig. 2b) and nucleated cells are selected based on DRAQ5 positivity to exclude the DNA negative cells (Fig. 2c). Given that the level of γ-H2AX in granulocytes is not markedly affected by radiation [36], lymphocytes are gated according to their area on bright field and side scatter for further measurement of the γ-H2AX fluorescence intensity and foci formation (Fig. 2d). Pan-nuclear γ-H2AX stained cells displayed a typical apoptotic pattern (Fig. 3a) which increased with time post irradiation (Fig. 3b), and were thus excluded from the γ-H2AX analysis. For each data point, 8273 ± 317 (mean ± SEM) cells were analyzed from 100 μL of whole blood within 1–2 min. Gamma H2AX yields were measured in 2076 ± 123 non-apoptotic lymphocytes.

**Fig. 4:**
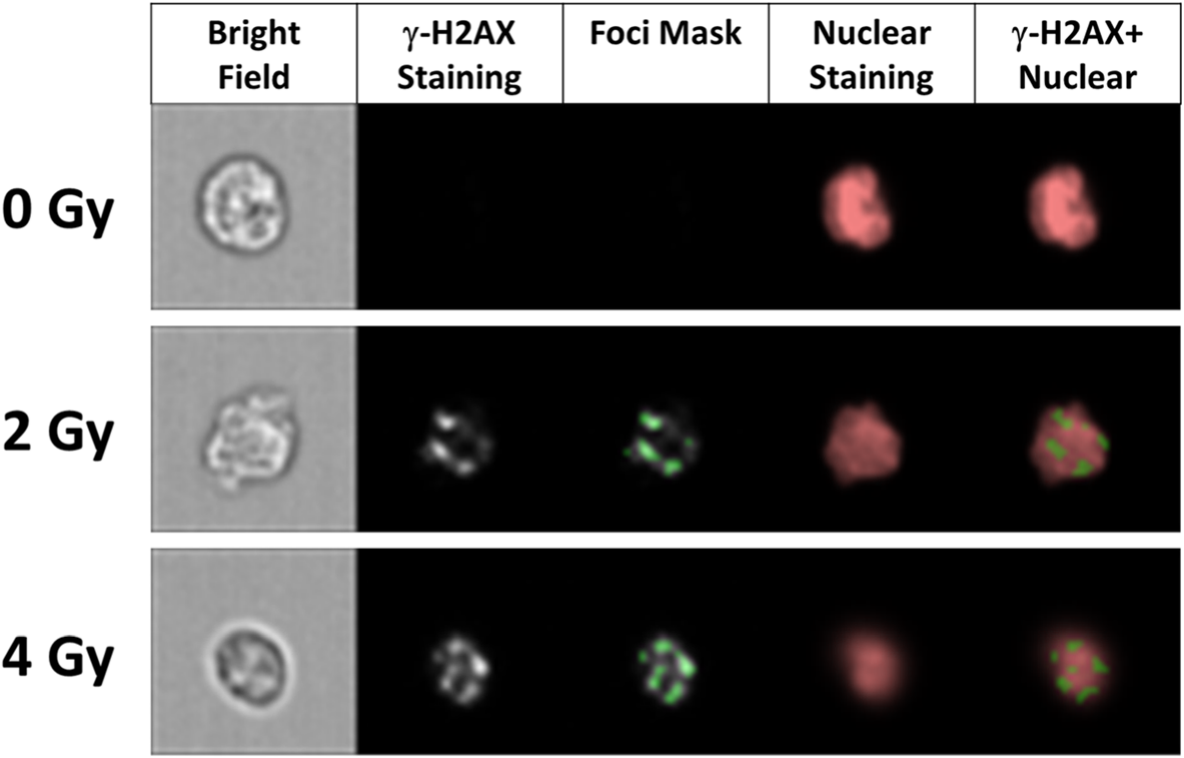
Representative images of γ-H2AX foci in human blood lymphocytes irradiated cells with γ-rays (0, 2 and 4 Gy), 1 h after irradiation. Cellular images displayed here show BF, γ-H2AX, γ-H2AX foci mask, DRAQ5 nuclear staining and a composite of γ-H2AX and DRAQ5. The spot counting wizard in the IDEAS® software was used to identify and enumerate γ-H2AX foci in all imagery (40x magnification)

The mean fluorescence intensity of γ-H2AX within nuclear boundary of individual cells was analyzed and exported from the IDEAS® software. The number of γ-H2AX foci was calculated using the spot counting wizard in IDEAS software as shown in Fig. 4. The wizard automatically creates masks based on subsets of cells by visual inspection (e.g. 30 low foci cells and 30 high foci cells – the selection of the cells was conducted by two independent investigators and reached consensus). This final spot mask is composed of three different functions on channel 2 and channel 5: (i) The Spot function identifies spots with a size< 1 pixel and a spot-to-background ratio greater than 4.5; (ii) The Peak function identifies intensity areas from an image with local maxima (bright spots) or minima (dark spots); (iii) The Range function identifies spots in the H2AX image with size < 200 pixels (50 μm^2^) and aspect ratios between 0 to 1; (iv) Overlapping with DRAQ5 image in channel 5. The representative foci mask is shown in Fig. 4. Finally, the Spot Count feature was calculated to enumerate foci identified by the mask. To test the accuracy of the foci counting, 100 cells were randomly selected and quantified for foci by visual inspection. The difference between the average number of foci by visual inspection and automated foci counting was 15.7% (0.63 foci ±0.07, mean ± SEM). A data analysis template file containing all required masks, features, plots and statistics was generated and applied to all samples using the batch processing option in IDEAS. Using the ISX, dose- and time-dependent γ-H2AX levels corresponding to radiation exposure were measured automatically over 24 h yielding an estimate of global DSB repair capacity as well as a measure of unrepaired DSBs.

### Dose response calibration curve

**Fig. 5:**
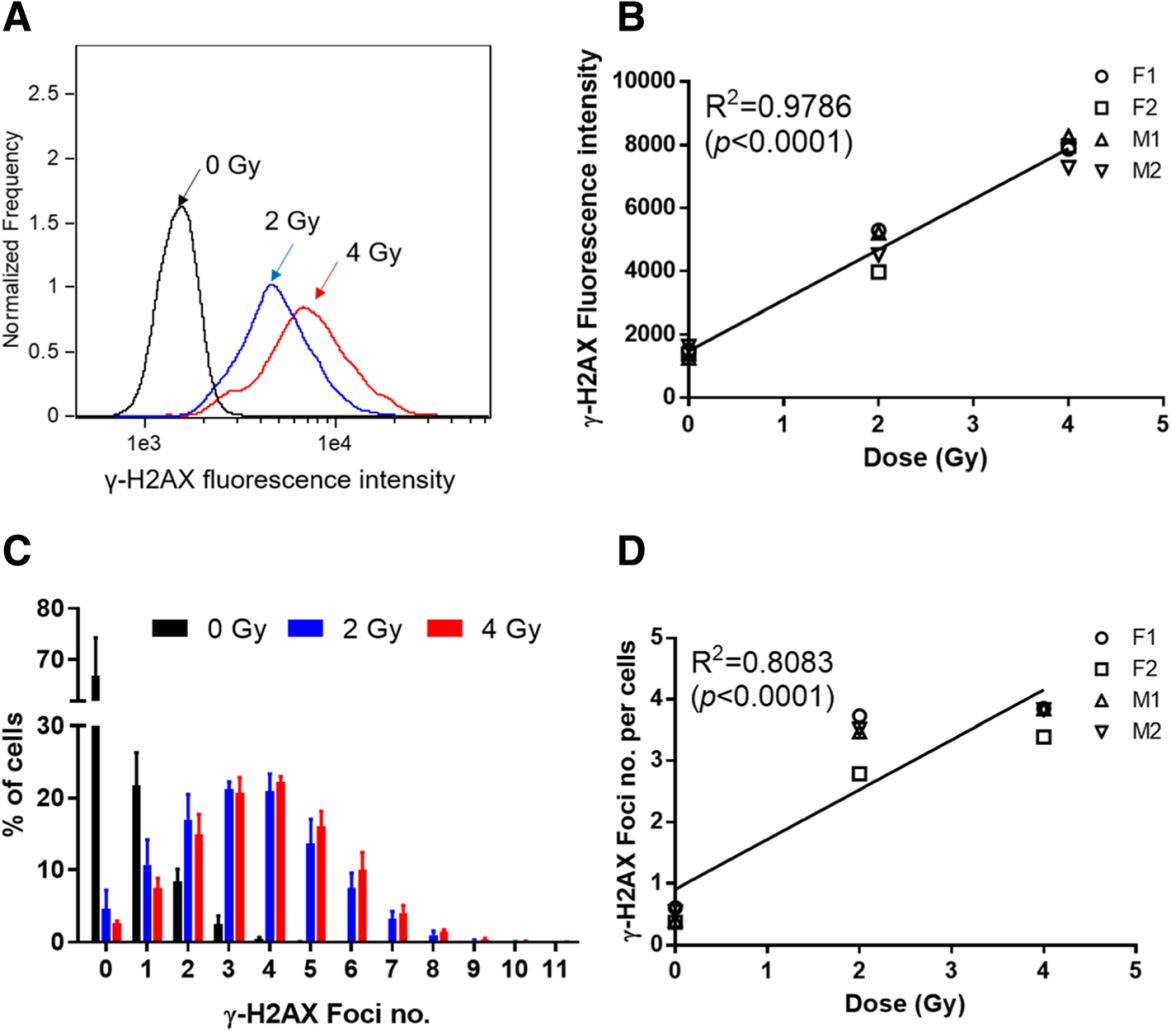
Dose-dependent changes of γ-H2AX in human blood lymphocytes 1 h after exposure with 4 Gy γ rays. **a** Representative distribution of γ-H2AX fluorescence intensity in lymphocytes from a female human donor, F1. **b** Radiation-induced changes in γ-H2AX mean fluorescence intensity in lymphocytes from 2 female and male donors, F1, F2, M1 and M2. **c** Distribution of cells with different numbers of γ-H2AX foci in lymphocytes from all donors (error bars represent the SEM). **d** Radiation-induced changes in γ-H2AX foci number from donors F1, F2, M1 and M2. Each symbol indicates averaged number of γ-H2AX foci for each donor; the fit represents the mean response

Figure 5 shows the average dose response for γ-H2AX fluorescence intensity and foci number obtained from 100 μL whole blood samples from four healthy donors, 1 h after 0, 2 and 4 Gy exposures. γ-H2AX intensity plots for non-irradiated human lymphocytes as well as samples irradiated with 2 Gy and 4 Gy γ rays show that γ-H2AX fluorescence intensity is highest in the 4 Gy irradiated cells, as expected (Fig. 5a). Figure 5b shows a linear increase of γ-H2AX fluorescence intensity with increasing radiation dose for the four human donors tested (*R*^2^ = 0.9786, *p* < 0.0001). The mean γ-H2AX foci distribution (Fig. 5c) indicates that the majority of the control, non-irradiated lymphocyte cells had 0 to 1 γ-H2AX foci, whereas the number of foci ranged from 0 to 8 in the irradiated cells. A small number of cells showed 8–10 differentiable foci after exposure to 4 Gy. The results also show that the linear fit for the mean number of γ-H2AX foci/cell increased up to 4 Gy (*R*^2^ = 0.8083, *p* < 0.0001, Fig. 5d), but the linearity was not as robust compared to mean γ-H2AX intensity levels.

### Measurement of γ-H2AX yields as a function of time following radiation exposure

**Fig. 6:**
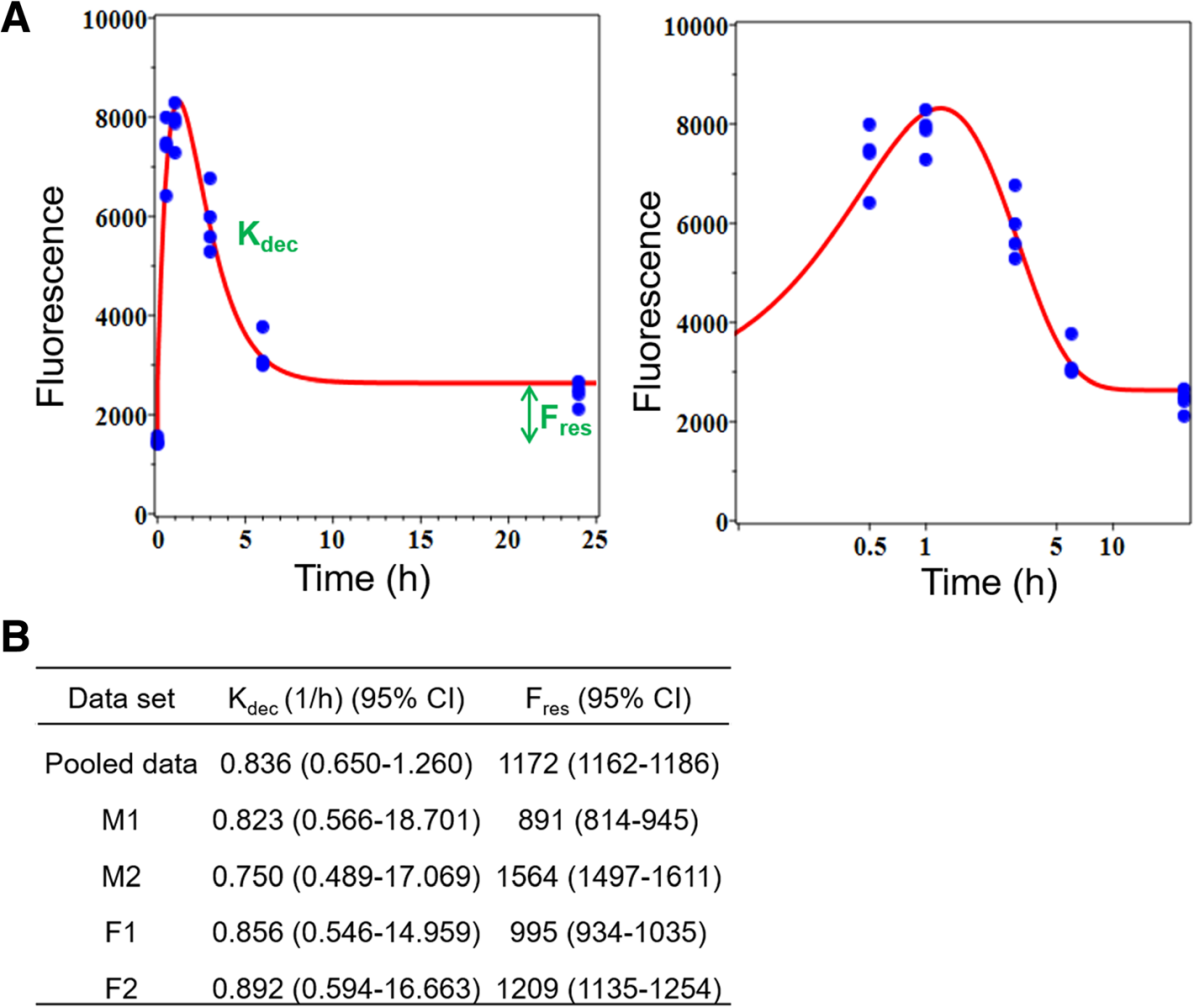
Time-dependent γ-H2AX fluorescence in human blood lymphocytes after 4 Gy irradiation. **a** Experimental data and model fit of γ-H2AX repair kinetics at 0.5, 1, 3, 6 and 24 h after ex vivo irradiation exposure are presented, based on mean fluorescence intensity; the right panel is zoomed in and plotted logarithmically to better visualize the details of the 0–12 h time-frame. **b** Each parameter of model fit of γ-H2AX repair kinetics was shown. K_dec_ is the constant for decay of γ-H2AX foci after irradiation. F_res_ is the residual value remaining at long times after irradiation

Figure 6a shows the time-dependent kinetics for each donor up to 24 h. It can be seen that radiation-induced γ-H2AX levels rapidly increased within 30 min and reached a maximum by ~ 1 h, after which time there was a rapid decline by 6 h, followed by a much slower rate of disappearance up to 24 h. The kinetics γ-H2AX data are presented using the mean fluorescence intensity measurements because the R^2^ coefficients showed a better fit for this approach, compared to mean foci levels, 0.5 to 24 h post-irradiation (Table 1).

**Table 1:** Dose response of γ-H2AX fluorescence and foci number at different time points

Time (h) after irradiation	γ-H2AX fluorescence intensity	γ-H2AX foci number per cell
	R^2^ (*p*-value)	R^2^ (*p*-value)
0.5	0.9705 (< 0.0001)	0.8295 (< 0.0001)
1	0.9786 (< 0.0001)	0.8083 (< 0.0001)
3	0.9533 (< 0.0001)	0.8170 (< 0.0001)
6	0.8994 (< 0.0001)	0.8540 (< 0.0001)
24	0.9017 (< 0.0001)	0.7473 (0.0003)

Figure 6b shows the data analysis for each individual on γ-H2AX yields as a function of time following radiation exposure. Two key parameters, the rate of decay (K_dec_) and the yield of residual unrepaired breaks (F_res_), were measured to define and quantify the γ-H2AX repair kinetics. Additional file 1 shows time-dependent response of γ-H2AX foci, 0.5 to 24 h post-irradiation. The data show that although the γ-H2AX foci time/dose-dependent repair pattern was similar to the fluorescence intensity endpoint, the foci data did not show any significant difference in repair capacity between the healthy donors.

## Discussion

Since it was first demonstrated by Rogakou, Bonner and colleagues that histone H2AX is rapidly phosphorylated on residue serine 139 in cells when DSBs are introduced into the DNA by ionizing radiation [37], the γ-H2AX assay has been widely used as a sensitive molecular marker of DNA damage and DSB repair capacity in a variety of human tissue and cell types [38, 39]. In recent years, the γ-H2AX biomarker has become a powerful tool to monitor DNA DSBs in translational cancer research with the potential to assess the radiosensitivity of prospective radiotherapy patients [5, 40]. The goal of the present work was to develop and optimize the γ-H2AX immunocytofluorescence protocol for high-content screening of double-stranded DNA breaks in finger-stick sized blood samples using IFC. The IFC technique allows fast and accurate analysis of γ-H2AX yields in several thousand cells per sample which would be extremely time consuming using conventional manual immunocytofluorescence protocols. In the present work, we have used our high-throughput IFC-based γ-H2AX assay to measure dose-dependent response and DSB repair kinetics in irradiated human blood samples.

To assess individual DSB repair capacity, radiation-induced γ-H2AX yields were measured for dose/time response in ex-vivo irradiated blood samples taken from four healthy donors (2 male, 2 female). Measurements of γ-H2AX fluorescence intensity and foci number at specific time points up to 24 h after exposure with 0, 2 and 4 Gy gamma rays showed a linear dose-dependent response and pattern of DNA repair, consistent with previous studies [10, 17, 20, 41]. The results highlight that the fluorescence intensity endpoint showed a better dose response compared to foci number given the small difference in foci number between 2 and 4 Gy. The reduced dose-response is likely attributed to the current configuration of our ISX IFC platform which only contains a 40x objective lens for image acquisition. The lower resolution of the 40x lens in comparison to a 60x objective is therefore likely to be responsible for the underestimation of γ-H2AX foci in the irradiated blood lymphocytes. Particularly in cells exposed to higher doses of radiation, there will be many γ-H2AX foci in close proximity to each other, leading to poor differentiation in smaller images with low spatial resolution. Recent studies by Durdik et al. [42] and Parris et al. [43] have shown that increasing the magnification from 40x to 60x together with extended depth of field (EDF) focus stacking option provided a more accurate assessment of foci number throughout the complete nuclear region in human lymphocytes exposed to low-dose ionizing radiation [42] and 2 Gy-irradiated immortalized fibroblasts [43]. Thus, these studies suggest that the 60x + EDF ISX configuration would permit enhanced foci identification thereby allowing better differentiation between the 2 and 4 Gy dose points, and identification of lower doses between 0 and 2 Gy. Further studies are necessary to address the assay dose limits for the sensitivity of the γ-H2AX foci and fluorescence intensity endpoints after ionizing radiation exposure and to expand this work to evaluate individual DNA repair capacity within a larger population.

Quantitative modeling of DNA repair kinetics based on fluorescence intensity showed that the decay constant of γ-H2AX foci after irradiation (K_dec_) was not markedly different among donors tested, whereas residual γ-H2AX fluorescence intensity (F_res_) was apparently higher in M2 and F2 than in the other two donors (M1 and F1), suggesting that M2 and F2 may have more unrepaired DSB 24 h after irradiation (Fig. 6b). The differences in DSB repair capacity between the 4 healthy donors tested here, show the potential of our high-throughput γ-H2AX assay to measure DNA repair kinetics on an individual-by-individual basis. Quantitative modeling of DNA repair kinetics based on foci number did not show any difference in DSB repair capacity between the four individuals (Additional file 1). This result was probably influenced by the visibly larger “scatter” in the foci data at 24 h, compared with the fluorescence intensity data at 24 h, widening the confidence intervals for F_res_ based on the foci data. Efforts to improve foci quantification with higher magnification and the use of EDF mentioned above, could enhance the quantification of DSB rejoining kinetics and assess the DSB repair capacity of specific individuals. Recent work by Kroeber et al. [23] showed the capability of the γ-H2AX assay to identify distinct outliers among a large cohort of 136 rectal cancer patients. They suggested that these patients are most probably radiosensitive and may have the highest risk of suffering radiotherapy-related late sequelae [23]. Interestingly, Yin et al. [8] recently reported enhanced DNA repair capacity in the peripheral blood mononuclear cells from a small cohort lung cancer patients tended to be associated with a poor response to radiation therapy, implicating a modulation of DNA repair [8].

It is known that the presence of γ-H2AX is not always linked specifically to DNA damage, but also to other cellular stages such as senescence, cell division or apoptosis [44]. In this case, the multi-spectral nature of IFC technology for γ-H2AX analysis would allow for the expansion to a quantitative multiplexed assay to analyze multiple radiation responsive biomarkers on a single cell. Also, the ability to target specific cell populations as well as eliminate interfering cells or debris will increase the number of cells that can be analyzed and potentially improve the sensitivity of the assay. In the current study, we measured γ-H2AX yields in focused DNA positive lymphocytes population instead of the total leukocytes. It is known that the sensitivity of lymphocytes and granulocytes to radiation are different whereby γ-H2AX levels in lymphocytes increased in a dose-dependent manner after 0–10 Gy γ-ray exposure, whereas levels in granulocytes were unaffected [36]. Further, residual levels of apoptosis in the irradiated samples are a potential confounding factor for the γ-H2AX total fluorescence analysis [45]. IFC image analysis using the IDEAS® software allowed us to automatically detect and eliminate pan-nuclear γ-H2AX stained lymphocytes based on fluorescence intensity and morphology. Pan-nuclear γ-H2AX response has been suggested as a biomarker to distinguish apoptotic cells from DNA damaged cells [46, 47]. We have shown here that the percentage of pan-nuclear γ-H2AX stained lymphocytes increased over time, up to 24 h after 4 Gy exposure (Fig. 3). These observations are consistent with other studies which show the apoptotic response of human lymphocytes upon radiation exposure [48–50].

Another advantage of our IFC-based γ-H2AX assay is both reduced assay time and time-to-result. First, our immunolabeling protocol presented here can be completed within 2 h, eliminating the need to prepare peripheral blood mononuclear cells which requires Ficoll gradient purification, an approach that is laborious and time-consuming, and will hamper large-scale population studies [51]. The IFC system is capable of acquiring cellular imagery at high flow rates from samples in suspension, reaching up to 1000 cells/s, making it faster than the automated microscopy systems and avoiding the need to create high quality slides [52].

Overall, further development and validation of the IFC-based γ-H2AX assay system presented in this work will allow for evaluation of DNA damage and DSB repair capacity with increased resolution, sensitivity, accuracy and high-speed image acquisition as compared to traditional flow cytometry and traditional microscope immunohistochemical methods [28, 30]. End-to-end automation of the IFC-based γ-H2AX assay can be achieved with the integration of our RABiT (Rapid Automated Biodosimetry Technology) platform for automated sample preparation from small volumes of blood [35]. Measurements of individual DSB repair capacity within a large population could offer valuable information to advance this high-throughput assay for translational research such as monitoring risk and response among radiotherapy patients.

## Conclusions

We have developed a high-throughput IFC-based γ-H2AX assay which is a faster and more efficient technique for assessing global DSB repair capacity. These studies could potentially pave the way for new individualized therapy approaches and new large-scale molecular-epidemiological studies, with the long-term goal of predicting individual radiosensitivity and risk of developing adverse effects related to radiotherapy treatment.

## Additional file

Additional file 1: Time-dependent γ-H2AX foci yields in human blood lymphocytes after 4 Gy irradiation. (A) Experimental data and model fit of γ-H2AX repair kinetics at 0.5, 1, 3, 6 and 24 h after ex vivo irradiation exposure are presented, based on foci number; the right panel is the zoomed picture for 0–12 h with a logarithmic time scale which helps to visualize early time points. (B) Each parameter of model fit of γ-H2AX repair kinetics was shown. K_dec_ is the constant for decay of γ-H2AX foci after irradiation. F_res_ is the residual value remaining at long times after irradiation. (DOCX 130 kb)

## Data Availability

The data that support the findings of this study are available from the corresponding author upon reasonable request.
