# Supplementary material for: Development of a high-throughput γ-H2AX assay based on imaging flow cytometry
Source: Radiat Oncol. 2019 Aug 22;14:150. doi: 10.1186/s13014-019-1344-7 (PMC6704696; doi:10.1186/s13014-019-1344-7)
Supplement: Supplementary file 1 — Time-dependent γ-H2AX foci yields in human blood lymphocytes after 4 Gy irradiation. (A) Experimental data and model fit of γ-H2AX repair kinetics at 0.5, 1, 3, 6 and 24 h after ex vivo irradiation exposure are presented, based on foci number; the right panel is the zoomed picture for 0–12 h with a logarithmic time scale which helps to visualize early time points. (B) Each parameter of model fit of γ-H2AX repair kinetics was shown. Kdec is the constant for decay of γ-H2AX foci after irradiation. Fres is the residual value remaining at long times after irradiation. (DOCX 130 kb) [file 13014_2019_1344_MOESM1_ESM.docx]

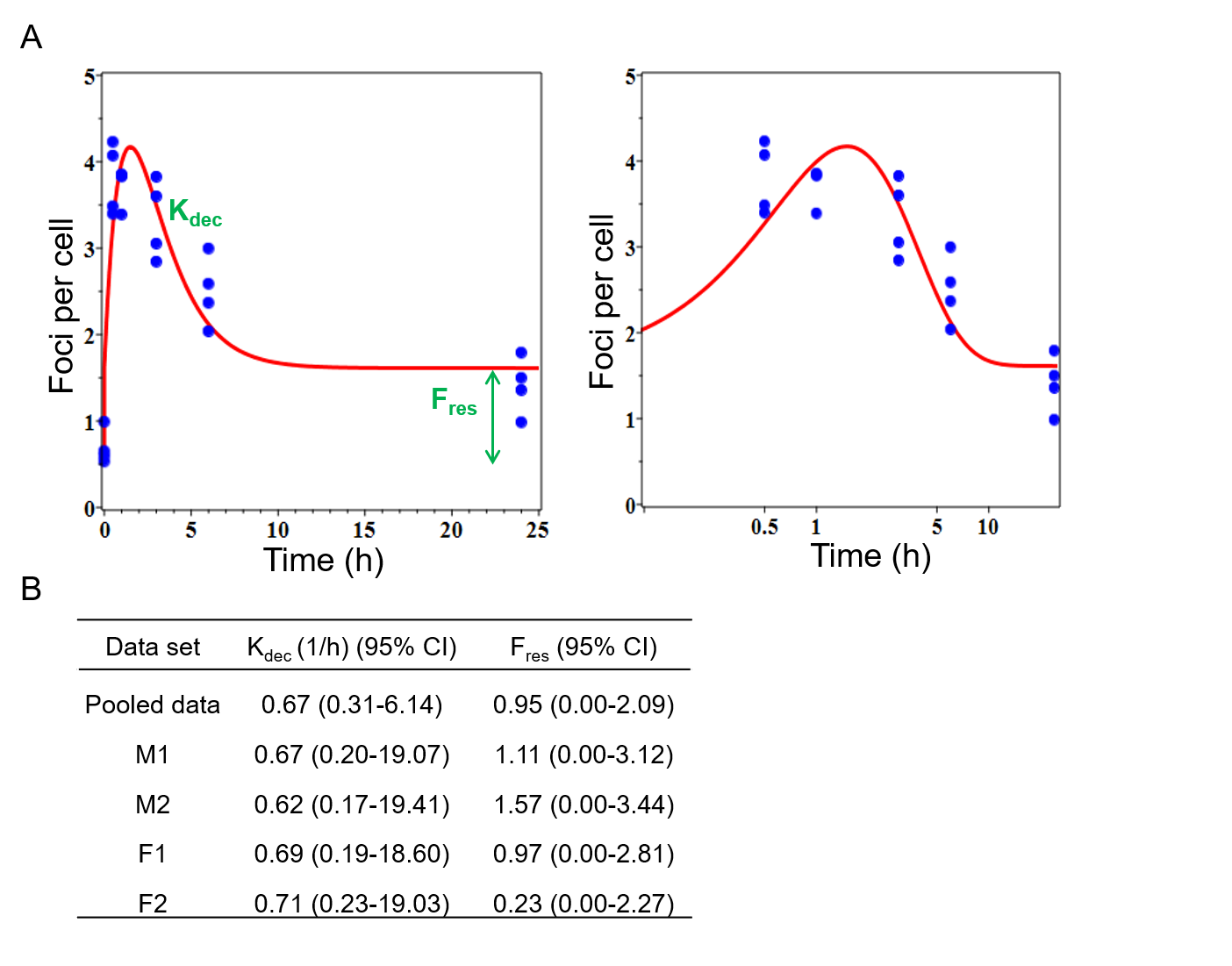


Additional File 1. **Time-dependent γ-H2AX foci yields in human blood lymphocytes after 4 Gy irradiation.** (A) Experimental data and model fit of γ-H2AX repair kinetics at 0.5, 1, 3, 6 and 24 h after *ex vivo* irradiation exposure are presented, based on foci number; the right panel is the zoomed picture for 0-12 h with a logarithmic time scale which helps to visualize early time points. (B) Each parameter of model fit of γ-H2AX repair kinetics was shown. K_dec_ is the constant for decay of γ-H2AX foci after irradiation. F_res_ is the residual value remaining at long times after irradiation.
